# Partial trisomy 19p13.3-p13.2 from antenatal diagnosis to 7-year follow-up: case report and literature review

**DOI:** 10.3389/fgene.2026.1869055

**Published:** 2026-10-22

**Authors:** Reema Abualnaja, Zohor Azher, Sarah Alkhaifi, Raghad Alkharouby

**Affiliations:** 1 College of Medicine, King Saud bin Abdulaziz University for Health Sciences, Jeddah, Saudi Arabia; 2 College of Medicine, Medical Genetics Department, Umm Al-Qura University, Makkah, Saudi Arabia; 3 Faculty of Nursing, King Abdulaziz University, Jeddah, Saudi Arabia

**Keywords:** chromosome 19, copy number variant, partial trisomy, trisomy, unbalanced translocation

## Abstract

Partial trisomy of the short arm of chromosome 19 (19p) is a rare chromosomal abnormality with significant clinical implications. We report the prenatal diagnosis and 7-year follow-up of a boy with partial trisomy 19p13.3-p13.2 and multiple congenital anomalies including microcephaly, unilateral hydronephrosis, mild bilateral hearing loss, failure to thrive, and global developmental delay. The pregnancy was complicated by antenatal findings of severe oligohydramnios and severe intrauterine growth restriction. Amniocentesis with chromosomal microarray analysis identified a 6.8 Mb copy number gain involving chromosome 19p13.3-p13.2, arr [GRCh37] 19p13.3-p13.2 (260,911_7,089,557)x3. Maternal cytogenetic analysis revealed an apparently balanced reciprocal translocation involving chromosome 13 and 19, supporting inheritance through unbalanced chromosomal segregation. The cytogenetic and clinical findings of our patient are discussed in this report. To our knowledge, this is the first report of partial trisomy 19p13.3–p13.2 as a result of maternal reciprocal translocation in the Middle East.

## 1 Introduction

Chromosomal abnormalities affect about 0.4%–0.9% of newborns worldwide, with nearly half of these cases presenting with congenital anomalies (Queremel Milan and Tadi, 2022). Chromosome 19 is one of the most gene-dense human chromosomes, and any defects involving this chromosome can lead to significant clinical manifestations (Puvabanditsin et al., 2009). Duplications of chromosome 19, whether partial or complete, has hardly been reported and are associated with a broad and variable phenotypic spectrum. The extent of the trisomic segment, as well as the genes and genetic components within it, contribute to the wide range of phenotypes observed in affected individuals.

Partial trisomy of the short arm of chromosome 19 (19p) was first reported by Byrne et al. in an infant with dysmorphic features, intrauterine growth restriction (IUGR), and mental disability among other significant clinical implications (Byrne et al., 1980). Since then, only a limited number of cases have been described in the literature worldwide (Puvabanditsin et al., 2009; Byrne et al., 1980; Andries et al., 2002; Salbert et al., 1992; Brown et al., 2000; Quigley et al., 2004; Stratton et al., 1995; Lybæk et al., 2009; Dolan et al., 2010; Siggberg et al., 2011; Lehman et al., 2011; Seidel et al., 2014; Ishikawa et al., 2013; Orellana et al., 2015; Nevado et al., 2015; Novikova et al., 2017; Martínez Anaya et al., 2020; Tenorio et al., 2020; Haber et al., 2021; Jouret et al., 2023). Studies investigating partial trisomy 19p have shed light on its impact on neurodevelopment and physical characteristics. Common clinical features reported include intellectual disability, delayed psychomotor development, growth retardation, craniofacial abnormalities, cardiac defects, and genitourinary anomalies.

In this report, we describe the prenatal diagnosis and 7-year longitudinal follow-up of a child with partial trisomy 19p13.3–p13.2 caused by a 6.83-Mb duplication involving chromosome 19p, resulting from unbalanced segregation of a maternally inherited balanced reciprocal translocation between chromosomes 13 and 19. The present case contributes to the limited literature on partial trisomy 19p by providing detailed cytogenetic characterization and exploring genotype–phenotype correlations based on the duplicated genomic interval and comparison with previously reported overlapping duplications.

## 2 Case description

The proband is a seven-year-old boy and the first child of healthy, non-consanguineous parents. His mother was a 33-year-old Middle Eastern woman with no significant past medical history, while his father was a healthy 34-year-old Native American man. The family history was unremarkable for congenital anomalies, neurodevelopmental disorders, recurrent pregnancy loss, or known chromosomal abnormalities.

The pregnancy course was without complications until 26 weeks of gestation, when routine obstetric ultrasonography demonstrated severe IUGR associated with marked oligohydramnios. Given the abnormal prenatal findings, amniocentesis was performed for genetic evaluation.

Chromosomal microarray analysis (CMA) identified a 6.83-Mb duplication involving chromosome 19p13.3–p13.2, reported as arr [GRCh37]19p13.3p13.2 (260,911_7,089,557)x3. Conventional fetal cytogenetic analysis with fluorescence *in situ* hybridization (FISH) demonstrated an unbalanced derivative chromosome 13 inherited from the mother and was reported as.

46, XY.ish der (13)t (13; 19) (p12; p13.2)mat (129F16/SP6+).

Parental chromosome studies subsequently identified a balanced reciprocal translocation in the mother.

46, XX,t (13; 19) (p12; p13.2).ish der (13) (129F16/SP6+),der (19) (129F16/SP6−,D19S238E+).

The fetal and maternal karyotypes are shown in Figures 1, 2, respectively.

**FIGURE 1:**
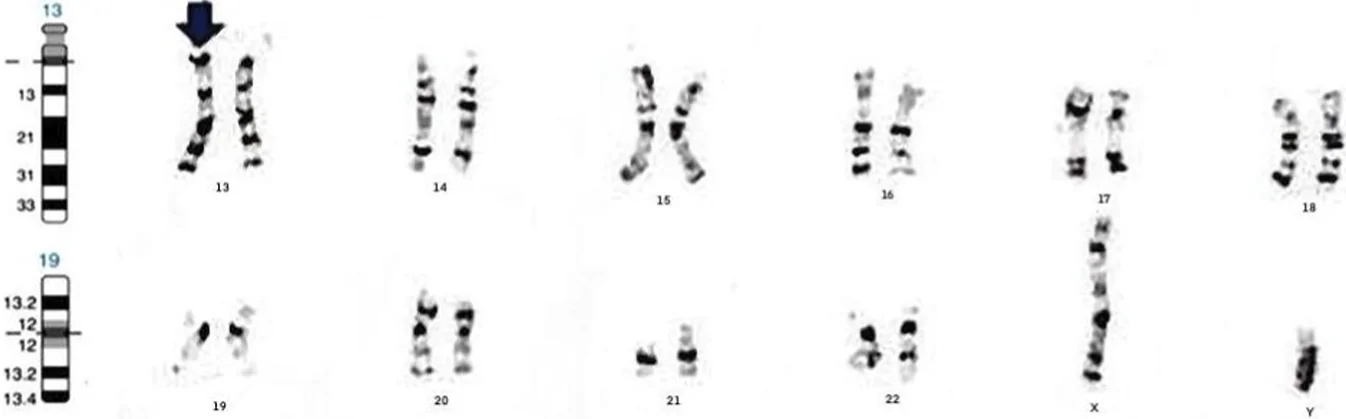
Fetal karyotype illustrating the abnormal male karyotype, with an unbalanced translocation involving the short arms of chromosome 13 and 19. Material from 19p has translocated to 13p, resulting in partial trisomy 19p13.2-pter.

**FIGURE 2:**
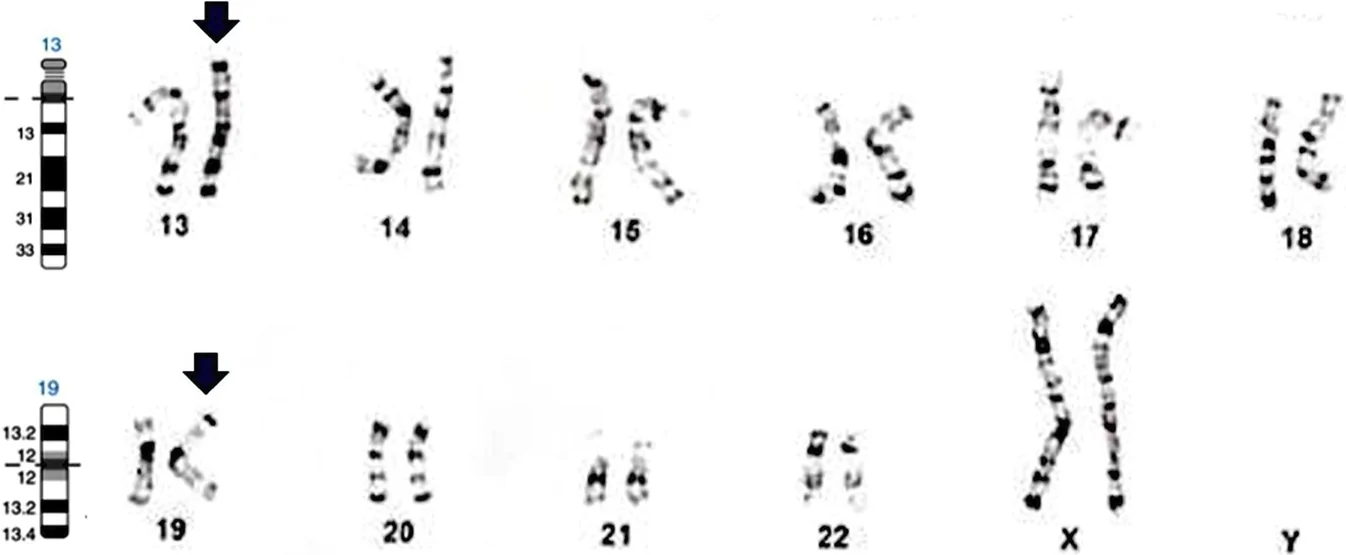
Maternal karyotype revealing that she is a carrier of a balanced t (13; 19) translocation.

The fetal chromosomal imbalance was therefore interpreted as partial trisomy of chromosome 19p13.3–p13.2 resulting from maternal transmission of an unbalanced derivative chromosome 13. The duplicated segment identified by chromosomal microarray corresponded to the additional chromosomal material detected by conventional cytogenetic analysis. No clinically significant loss of chromosome 13 material was detected, indicating that the patient’s genomic imbalance was limited to duplication of chromosome 19p13.3–p13.2. A summary of the genetic characterization methods is provided in Supplementary Data sheet.

Following comprehensive genetic counseling, the antenatal findings, anticipated clinical uncertainties, and available pregnancy management options were discussed with the family. After careful consideration and in accordance with their personal values and beliefs, the parents elected to continue the pregnancy. The pregnancy was subsequently monitored with serial ultrasonographic assessments, which demonstrated persistent fetal growth restriction throughout the remainder of the pregnancy. The umbilical cord was also noted to contain a single umbilical artery.

## 3 Diagnostic assessment

### 3.1 Neonatal findings and birth anthropometry

The infant was delivered spontaneously at 38 weeks of gestation. Birth weight was 1,110 g, consistent with severe growth restriction. The Apgar score was 7 at 1 minute. Shortly after birth, he developed respiratory distress requiring high-flow nasal cannula support and nasogastric tube feeding. Placental examination demonstrated reduced placental size with otherwise normal morphology. The neonate was admitted to the neonatal intensive care unit (NICU), where he remained for close monitoring for 11 days before being discharged home with ongoing multidisciplinary follow-up.

Physical examination at birth demonstrated severe growth restriction with microcephaly, enlarged, soft, and full fontanelles, sparse and arched eyebrows, hypertelorism with upslanting palpebral fissures, low-set ears, a prominent nose with low-hanging columella, a long smooth philtrum with thin lips, and mild microretrognathia.

Cranial ultrasonography identified a small left choroid plexus cyst without additional structural brain parenchymal abnormalities. The cyst resolved spontaneously by 6 months of age. Newborn hearing screening was abnormal, and confirmatory auditory brainstem response testing performed 2 weeks later demonstrated mild-to-moderate bilateral conductive hearing loss.

Renal ultrasonography demonstrated mild left-sided hydronephrosis. Voiding cystourethrography subsequently identified vesicoureteral reflux (grade III on the left and grade I on the right). Cardiac evaluation by echocardiography revealed no structural cardiac abnormalities.

### 3.2 Infancy and early childhood clinical course

Feeding difficulties were evident immediately after birth and persisted beyond the neonatal period. Nasogastric tube feeding continued throughout the NICU admission and for approximately 2 months after discharge. Despite gradual improvement in oral intake, the patient continued to experience dysphagia with both solids and liquids, confirmed by a formal swallowing assessment. Gastroesophageal reflux disease developed during infancy and was managed with metoclopramide therapy for approximately 2 years.

During early childhood, the patient experienced recurrent episodes of febrile seizures beginning in infancy and continuing until approximately 5 years of age, resulting in multiple hospital admissions. Serial electroencephalograms (EEGs) remained normal, and long-term antiseizure medication was not indicated. At 4 years of age, mild lower-limb spasticity became clinically apparent. Further evaluation with brain magnetic resonance imaging and computed tomography showed no structural brain abnormalities. Persistent sleep disturbances were also reported despite normal polysomnography.

The patient experienced recurrent otitis media requiring tympanostomy tube insertion and subsequently developed chronic otomycosis. At 4 years of age, clinical examination identified a soft cleft palate associated with a bifid uvula.

Orthopedic evaluation demonstrated bilateral positional congenital foot deformities, which were successfully corrected using orthotic management over a 3-year period.

The patient also experienced recurrent urinary tract infections during early childhood. Vesicoureteral reflux and hydronephrosis resolved spontaneously by 3 years of age, and urinary tract infections became less frequent following circumcision.

### 3.3 Developmental milestones

Global developmental delay became apparent during infancy and affected all developmental domains. Gross motor development was markedly delayed. Head control was achieved at approximately 5 months of age, independent sitting at 16 months, rolling from prone to supine at 18 months, and independent walking at 3 years of age. Fine motor skills were also delayed. The patient was able to reach and transfer objects at approximately 9 months of age but has yet to develop a mature pincer grasp.

Language development remains profoundly impaired. At 7 years of age, he continues to vocalize and babble during play but has not developed intelligible speech. Communication is primarily nonverbal, relying on gestures, such as pointing, to express his needs.

Social development is also significantly affected. He demonstrates poor eye contact, inconsistent responses to caregivers, limited comprehension of verbal commands, and frequent behavioral outbursts, including episodes of head banging when frustrated or attempting to communicate.

### 3.4 Clinical status at seven years of age

At the most recent clinical assessment, the patient remained significantly developmentally delayed and fully dependent on his caregivers for activities of daily living, including feeding, dressing, hygiene, and toileting. His growth parameters remained markedly reduced, with weight, height, and head circumference all below the 3^rd^ centile.

Physical examination demonstrated persistent microcephaly with a relatively broad forehead and dysmorphic craniofacial features. These included arched eyebrows, hypertelorism with upslanting palpebral fissures, intermittent esotropia, a broad nasal bridge with a prominent nasal tip and low-hanging columella, prominent low-set ears, a long smooth philtrum, a thin upper vermilion border, and mild microretrognathia. No clinical photographs are included, in accordance with parental preference.

Neurological examination showed axial hypotonia with mild appendicular spasticity. He was ambulatory but had significant global developmental delay, most prominently affecting expressive language, adaptive functioning, and social communication. He remained nonverbal and communicated primarily through gestures to his caregivers. The remainder of the systemic examination was unremarkable. He continues to receive multidisciplinary follow-up, including speech and language pathology, occupational rehabilitation, and physiotherapy.

## 4 Discussion

Partial trisomy of chromosome 19p is an exceptionally rare chromosomal disorder with substantial clinical heterogeneity. Since the first description of partial trisomy 19p, only a limited number of patients with molecularly characterized duplications have been reported, making genotype–phenotype correlation challenging because of differences in duplication size, breakpoint location, associated chromosomal rearrangements, and methods of genomic characterization. The present case expands the clinical spectrum by describing the prenatal diagnosis and 7-year longitudinal follow-up of a child with a molecularly confirmed duplication of chromosome 19p13.3–p13.2. The combination of prenatal diagnosis, detailed cytogenetic characterization, long-term follow-up, and comparison with previously reported patients provides additional insight into the clinical manifestations associated with this duplicated interval (Puvabanditsin et al., 2009; Byrne et al., 1980; Andries et al., 2002; Salbert et al., 1992; Brown et al., 2000; Quigley et al., 2004).

### 4.1 Comparison with previously published cases

Supplementary Table S1 summarizes previously published reports of pure, non-mosaic chromosome 19p duplications. Mosaic cases and cases with additional chromosomal imbalances were excluded to allow a more comparable genotype–phenotype discussion with the present patient.

Compared with previously published cases, our patient demonstrates many of the core clinical manifestations that appear to be consistently associated with duplications involving distal chromosome 19p (Supplementary Table S1). Neurodevelopmental impairment was the most consistent finding across the published literature, regardless of duplication size or mechanism of inheritance, although the severity varied from mild learning difficulties (Andries et al., 2002; Siggberg et al., 2011; Nevado et al., 2015) to severe global developmental delay (Puvabanditsin et al., 2009; Byrne et al., 1980; Salbert et al., 1992). Similarly, our proband exhibited severe global developmental delay affecting gross motor, fine motor, language, adaptive and social skills. At 7 years of age, expressive language remains extremely limited, and he continues to require assistance with all activities of daily living. The prolonged clinical follow-up available in the present case provides valuable information regarding long-term functional outcome data that are unavailable in many earlier reports limited to infancy or early childhood. Additionally, prenatal or postnatal growth restriction, microcephaly, and characteristic craniofacial dysmorphism, including hypertelorism, low-set ears, philtral abnormalities, and cranial shape abnormalities, were repeatedly observed among individuals with overlapping duplications and were also present in our patient. These findings suggest that although the duplicated genomic intervals vary considerably among reported patients, a recognizable neurodevelopmental and craniofacial phenotype is shared by many individuals with duplications involving chromosome 19p13 (Puvabanditsin et al., 2009; Byrne et al., 1980; Andries et al., 2002; Salbert et al., 1992; Brown et al., 2000; Quigley et al., 2004; Stratton et al., 1995; Lybæk et al., 2009; Dolan et al., 2010; Siggberg et al., 2011; Lehman et al., 2011; Seidel et al., 2014; Ishikawa et al., 2013; Orellana et al., 2015; Nevado et al., 2015; Novikova et al., 2017; Martínez Anaya et al., 2020; Tenorio et al., 2020; Haber et al., 2021; Jouret et al., 2023).

In contrast to the relatively consistent neurodevelopmental phenotype, involvement of other organ systems demonstrated substantial inter-individual variability. Cardiac abnormalities, for example, have been described in several published patients, including septal defects and more complex congenital heart disease (Salbert et al., 1992; Stratton et al., 1995; Seidel et al., 2014; Martínez Anaya et al., 2020), whereas echocardiography in our patient demonstrated no structural cardiac abnormality. Similarly, musculoskeletal manifestations ranged from isolated hypotonia to limb anomalies, scoliosis, and congenital foot deformities. Our patient has bilateral positional foot deformities and mild lower-limb spasticity without major skeletal malformations.

Hearing impairment and recurrent otolaryngological disease were reported less consistently among published patients but represent another area of phenotypic overlap. Conductive or sensorineural hearing loss has been documented in several previously reported individuals (Salbert et al., 1992; Quigley et al., 2004; Seidel et al., 2014; Tenorio et al., 2020; DECIPHERForeman, 2023), whereas recurrent otitis media was reported only sporadically. Our patient demonstrated mild-to-moderate bilateral conductive hearing loss detected during the neonatal period together with recurrent otitis media requiring tympanostomy tube placement. Likewise, feeding difficulties, gastroesophageal reflux, and failure to thrive have been described in several reports (Supplementary Table S1) and closely parallel the persistent feeding difficulties, dysphagia, and prolonged gastroesophageal reflux observed in the present patient.

Renal and genitourinary abnormalities appear to represent a recurrent, although less frequent, component of the phenotype. Hydronephrosis, urinary tract abnormalities, cryptorchidism, and other genitourinary anomalies have each been reported in isolated patients within the published literature. Our patient presented with unilateral hydronephrosis, bilateral vesicoureteral reflux, and recurrent urinary tract infections during early childhood. Although these findings resolved or improved over time, their recurrence in the present case and in previously reported patients suggests urinary tract involvement as part of the variable phenotypic spectrum of distal chromosome 19p duplications.

Neurological manifestations also varied considerably among reported patients. Hypotonia was commonly described, whereas seizures were reported less consistently. In our patient, the seizure history was limited to recurrent febrile seizures with normal serial electroencephalograms, normal neuroimaging, and no requirement for long-term antiseizure therapy. Distinguishing recurrent febrile seizures from epilepsy is clinically relevant because the available literature suggests that seizure disorders are variable rather than universal manifestations of chromosome 19p duplication, and the neurological prognosis should therefore be individualized according to each patient’s clinical course rather than inferred solely from the chromosomal abnormality.

### 4.2 Comparison with DECIPHER cases

To further explore genotype–phenotype correlations, we reviewed the DECIPHER database for cases carrying duplications entirely contained within the duplicated genomic interval identified in our patient, GRCh37: chr19:260,911–7,089,557 (Supplementary Table S2). This inclusion criterion was selected to assess whether smaller overlapping duplications within the same interval shared elements of the phenotype observed in our patient, rather than to define a minimal critical region.

The DECIPHER cases further illustrate the marked phenotypic heterogeneity associated with duplications involving chromosome 19p13. Although all included duplications were contained within our patient’s duplicated interval, duplication size ranged from several kilobases to more than 4 Mb and was associated with variable clinical presentations. Developmental delay was the most frequently reported feature, supporting the observation from the published literature that neurodevelopmental impairment represents the most consistent clinical manifestation of chromosome 19p duplications. Other reported features, including autism spectrum disorder, intellectual disability, feeding difficulties, failure to thrive, craniofacial abnormalities, and short stature, were observed only in subsets of patients, reflecting substantial variability despite overlapping genomic coordinates.

Comparison of the published literature and the DECIPHER cohort suggests that the extent of genomic overlap alone does not fully explain phenotypic severity. Several individuals carrying relatively small duplications within the interval presented with developmental delay or isolated neurodevelopmental disorders, whereas others with substantially larger duplicated segments demonstrated multisystem involvement affecting growth, hearing, craniofacial development, gastrointestinal function, and the genitourinary system. These findings suggest that clinical severity should be interpreted in the context of gene content, chromosomal breakpoint location, rearrangement mechanism, inheritance pattern, and additional genetic or environmental modifiers.

At present, the available evidence remains insufficient to define a single critical region responsible for the phenotype or to establish a robust correlation between duplication size and clinical severity. Consequently, accurate phenotypic prediction for newly diagnosed patients remains challenging, emphasizing the importance of individualized clinical assessment and long-term multidisciplinary follow-up.

### 4.3 Gene-level analysis of the duplicated interval

Gene-level analysis of the 19p13.3–p13.2 duplicated region was performed using the DECIPHER database. This review identified several morbid genes with established disease associations that may be relevant to our patient’s phenotype (Supplementary Table S3).

A number of genes in this region are linked to neurological or developmental disorders. DOT1L is associated with a neurodevelopmental disorder with intracranial anomalies, while SEMA6B, KDM4B, and DOHH are associated with neurodevelopmental disorders. TUBB4A is linked to neurological disorders, including hypomyelination with atrophy of the basal ganglia and cerebellum. FZR1 has been associated with intellectual disability and epilepsy, and LMNB2 with a disorder that includes microcephaly. These genes are relevant candidates for the severe developmental delay and microcephaly seen in the present patient, although causality cannot be established at the level of individual genes.

Beyond neurodevelopment, the affected region also contains genes associated with hearing, mitochondrial function, immune function, hematological disease, and other systemic phenotypes. These associations may be relevant to the patient’s conductive hearing loss, recurrent otolaryngological infections, feeding difficulties, poor growth, and genitourinary involvement. However, the overall clinical presentation is unlikely to be explained by a single gene and may instead reflect the cumulative effect of increased dosage across multiple genes within the duplicated segment.

We also reviewed DECIPHER’s dosage-related and gene constraint metrics to identify genes with predicted sensitivity to copy number gain. The pTriplo score estimates the probability that a gene may be triplosensitive, with higher values indicating greater predicted sensitivity to an additional copy. DECIPHER considers values ≥ 0.68 to correspond to a predicted odds ratio of at least 2, while values ≥ 0.94 correspond to a predicted odds ratio of at least 2.7. These values are useful for prioritizing candidate genes, but they remain predictive rather than definitive measures of dosage pathogenicity.

Among the 51 morbid genes analyzed, 21 had pTriplo scores ≥0.68. Nine genes had particularly high scores of ≥0.94: TUBB4A (1.00), MAP2K2 (1.00), CLPP (0.98), DPP9 (0.98), FZR1 (0.98), GPX4 (0.97), EEF2 (0.97), KDM4B (0.96), and AP3D1 (0.94). The presence of multiple genes with elevated predicted dosage sensitivity supports the possibility that increased copy number contributes to the observed phenotype through a multigene effect.

Nevertheless, several limitations should be considered when interpreting these candidate genes. Although several genes within the duplicated region are associated with phenotypic features that overlap with our patient’s presentation, ClinGen has not established definitive triplosensitivity for these genes. Additionally, some reported gene-disease associations result from mechanisms other than increased copy number, such as loss-of-function or biallelic variants. Therefore, the presence of a gene within the duplicated interval alone cannot establish a causal relationship with the corresponding phenotype.

Overall, this gene-level analysis complements the comparison with published and DECIPHER cases by demonstrating that the duplicated 19p13.3–p13.2 interval contains multiple genes with clinically relevant disease associations and variable levels of predicted dosage constraint. These findings support the concept that the phenotype of partial trisomy 19p is likely to reflect the combined effect of multiple genes within the duplicated segment rather than a single causative gene. The current evidence remains insufficient to establish a definitive gene-level genotype–phenotype relationship, and further accumulation of molecularly characterized cases with detailed phenotyping will be required to determine whether specific genes or gene combinations consistently contribute to the clinical manifestations of 19p duplications.

### 4.4 Clinical implications for diagnosis and genetic counseling

This case highlights the complementary role of chromosomal microarray and cytogenetic testing in the diagnosis of rare chromosomal duplications. Chromosomal microarray detected the 19p13.3–p13.2 copy number gain, while fetal and parental cytogenetic studies identified the underlying chromosomal rearrangement. These complementary approaches are important to further classify chromosomal abnormalities. In clinical practice, copy number analysis identifies the genomic imbalance, while karyotyping and targeted cytogenetic testing can determine whether the rearrangement is *de novo* or inherited. This distinction is important for recurrence risk assessment, reproductive counseling, and consideration of testing for family members.

Genetic counseling should emphasize that partial trisomy 19p is associated with a variable phenotype. Neurodevelopmental impairment, growth restriction, microcephaly, and craniofacial dysmorphism appear recurrent, but involvement of other organ systems varies between patients. Counseling should therefore be individualized and should address uncertainty in prognosis, the need for multidisciplinary follow-up, and the importance of parental cytogenetic testing when an unbalanced rearrangement is identified.

### 4.5 Strengths and limitations

The main strength of this report is the combination of prenatal diagnosis, molecularly defined 19p13.3–p13.2 duplication, cytogenetic characterization of the underlying rearrangement, and longitudinal follow-up to 7 years of age. This provides clinically useful information regarding developmental trajectory, functional outcome, and multisystem involvement.

The limitations are those inherent to a single case report. Causality between individual duplicated genes and specific clinical features cannot be established. In addition, genotype-phenotype comparisons are limited by heterogeneity among previously reported cases, including differences in genomic testing methods, duplication size, breakpoint resolution, associated chromosomal rearrangements, and variable phenotypic reporting. DECIPHER-based comparisons are also limited by incomplete clinical information and ascertainment bias.

## 5 Conclusion

This case expands the clinical and cytogenetic spectrum of partial trisomy 19p13.3–p13.2 and provides long-term follow-up from antenatal diagnosis to 7 years of age. The findings support a recurrent phenotype characterized by neurodevelopmental impairment, growth restriction, microcephaly, and distinctive craniofacial features, with variable multisystem involvement. The case also emphasizes the importance of parental cytogenetic testing, individualized genetic counseling, and long-term multidisciplinary follow-up for patients with rare chromosomal duplications.

## Data Availability

The original contributions presented in the study are included in the article/Supplementary Material, further inquiries can be directed to the corresponding author.

## Supplementary material

SUPPLEMENTARY TABLE S1 Summary of clinical characteristics reported in published cases of pure 19p13.3 duplications.

SUPPLEMENTARY TABLE S2 DECIPHER copy number variants entirely contained within the duplicated interval identified in the present patient and their associated phenotypes.

SUPPLEMENTARY TABLE S3 DECIPHER morbid genes within the duplicated 19p13.3–p13.2 interval.
