# Supplementary material for: Partial trisomy 19p13.3-p13.2 from antenatal diagnosis to 7-year follow-up: case report and literature review
Source: Front Genet. 2026 Oct 22;17:1869055. doi: 10.3389/fgene.2026.1869055 (PMC13734668; doi:10.3389/fgene.2026.1869055)
Supplement: Supplementary file 1 [file DataSheet1.pdf]

Genetic Characterization of the Fetus and Mother

Genetic characterization was performed using conventional karyotyping, metaphase fluorescence in situ hybridization (FISH), and chromosomal microarray analysis (CMA). Prenatal testing was performed on an amniotic fluid specimen from the fetus, while maternal chromosome analysis was performed on a constitutional blood specimen.

For the fetal sample, conventional karyotyping was performed on cultured amniocytes using GPC banding at a reported resolution of 450 bands. Fifteen metaphases were counted, five were analyzed, and three were karyotyped. Metaphase FISH was subsequently performed using the ToTelVysion subtelomere probe cocktail 14 (Abbott Molecular), targeting the19p subtelomeric region, with control regions on 19p13 and the 19q subtelomeric region.

Prenatal CMA was performed on uncultured amniotic fluid using the Genome-Wide SNP Array CytoScan HD (Affymetrix), containing approximately 2.6 million markers, including 750,000 SNPs and 1.9 million non-polymorphic probes. Data were analyzed using Affymetrix Chromosome Analysis Suite (ChAS) version 3.1.0.15, with genomic coordinates based on the GRCh37/hg19 genome build.

DNA fingerprint analysis was performed on maternal blood and fetal aminocytes using PCR amplification of short tandem repeat loci followed by capillary electrophoresis to distinguish sample origins and assess for cellular mixture.

Fot the maternal sample, conventional karyotyping was performed on constitutional blood using GPG banding at a reported resolution of 550 bands. Twenty metaphases were counted, six were analyzed, and three were karyotyped. Metaphase FISH was performed using the same ToTelVysion subtelomere probe cocktail 14 (Abbott Molecular) used for fetal testing.
